# Binge Eating and Compulsive Buying During Cabergoline Treatment for Prolactinoma: A Case Report

**DOI:** 10.3389/fpsyt.2022.844718

**Published:** 2022-05-26

**Authors:** Ana Carolina Correa e Castro, Andressa Alexandre de Araujo, Mariana Coelho Botelho, João Bosco Nascimento, Rafaela Marchon de Souza, Monica Roberto Gadelha, Antonio E. Nardi, Alice Helena Dutra Violante

**Affiliations:** ^1^Institute of Psychiatry, Federal University of Rio de Janeiro, Rio de Janeiro, Brazil; ^2^Federal University of Rio de Janeiro, Rio de Janeiro, Brazil; ^3^UFRJ Scientific Initiation Program (a) and Rio de Janeiro State Research Foundation (b), Rio de Janeiro, Brazil; ^4^Endocrinology, Federal University of Rio de Janeiro School of Medicine, Rio de Janeiro, Brazil; ^5^Full Professor of Psychiatry, Federal University of Rio de Janeiro School of Medicine, Instituto de Psquiatria da Federal University of Rio de Janeiro, Rio de Janeiro, Brazil

**Keywords:** prolactinoma, cabergoline, impulsivity, compulsive behavior, case report

## Abstract

Prolactinomas are the most prevalent functional pituitary adenomas. They are usually treated clinically with dopamine agonists. The most widely used and suitable drug is cabergoline (CAB), a specific D2 dopamine agonists. Patients in prolactinoma treatment with CAB commonly report physical side effects, but aberrant behavioral changes such as increased impulsivity have also been reported recently. We report the case of a 47-year-old Brazilian woman with prolactinoma that developed compulsive buying, binge eating, and hypersexuality after four years of CAB treatment. In her psychiatric evaluation, the patient scored high levels on the following scales: Compulsive Buying Scale (CBS), Binge Eating Scale (BES), and Barratt Impulsiveness Scale-11 (BIS11). She also reported financial problems and weight gain in addition to her social and clinical problems. Impulsivity disorders may appear with the use of CAB and other dopamine agonists. We suggest that more observational studies with a large patient sample and specific regular psychiatric evaluations during treatment are necessary for patients in use of CAB, especially those treated for several years.

## Introduction

Prolactinomas are the most common functional pituitary adenomas, representing 60% of all clinically evident pituitary tumors (1). The first line of prolactinoma treatment is the use of dopamine agonists (2). Bromocriptine (BRC) and cabergoline (CAB) are widely used ergot-derived dopamine agonists. Quinagolide is also prescribed but is not available in Brazil (2), dopamine agonists therapy normalizes prolactin (PRL) levels in most cases, with resolution of gonadal dysfunction and infertility, besides tumor shrinkage. Dopamine agonists are generally well-tolerated, but in some cases side effects such as nausea, vomiting, nasal congestion, postural hypotension, dizziness, and syncope can occur. Rhinorrhea, painless vasospasm, pleural effusion, pulmonary or retroperitoneal fibrosis, insomnia, mood changes, and psychosis may also be reported, while the increased risk of valvular heart disease is still controversial (2, 3).

Impulse control disorder (ICD) is described as a “failure to resist an impulse, drive, or temptation to perform an act that is harmful to the person or others,” according to the Diagnostic and Statistical Manual of Mental Disorders (DSM5) criteria. Pathological gambling, hypersexuality, binge eating, and compulsive shopping are included among ICDs in accordance with DSM5, even though they are classified in different DSM5 categories (4).

In addition to prolactinomas, dopamine agonists therapy can also be used in acromegaly, growth hormone secreting pituitary adenoma, Parkinson’s disease (PD), and restless legs syndrome, and among these diseases, ICD is reported mainly in prolactinoma. (5) An association between dopamine agonists use and impulsivity in hyperprolactinemia was first published in 2007. Davie et al. reported on a 38-year-old woman with a microprolactinoma who developed pathologic gambling one year after initiating CAB. In 2009, Falhammar reported a second case of ICD in a 50-year-old man with a microprolactinoma treated with CAB with pathologic gambling and hypersexuality, despite low testosterone level (6).

Subsequent years witnessed an increase in case reports of dopamine agonists -induced ICDs in patients with prolactinoma, despite low doses of dopamine agonists in these patients compared to PD and restless legs syndrome. Dopamine agonists-induced ICD was assessed in many series and meta-analyses mainly in PD, but few studies in prolactinoma and only a handful with a cross-sectional design (5–9).

## Case Description

In 1995, a 21-year-old female patient presented galactorrhea associated with menstrual irregularity and sought medical attention. She was diagnosed with hyperprolactinemia, started BRC to control serum PRL levels, but after one month she interrupted use on her own due to gastrointestinal intolerance (vomiting) and dizziness. Approximately one year later, her initial symptoms persisted, and she resumed therapy with BRC (maximum daily dose 3.5 mg), suspended after a few months due to pregnancy, which evolved without complications. Her first magnetic resonance image (MRI) in 1996 before her first pregnancy revealed a lesion less than 5 mm in diameter in the anterior pituitary gland.

At 24 years of age, the patient appeared for medical treatment at the Endocrinology Clinic of the Clementino Fraga Filho University Hospital (HUCFF-UFRJ) in Rio de Janeiro. Her main complaints were severe headache, altered visual acuity, and amenorrhea. On physical examination, she presented galactorrhea and altered field of vision (left temporal hemianopsia and loss of upper right temporal and nasal fields). In February 1999, a new MRI revealed possible pituitary apoplexy and a 1.3 mm × 1.0 mm lesion in the right anterior pituitary, with contralateral shift of the infundibulum. She was not on dopamine agonists therapy at the time.

After this assessment, the decision was made to reintroduce BRC therapy, which was maintained until 2000 when the patient entered her second pregnancy, again without complications. Her visual campimetry improved considerably while under treatment, with good control of her PRL level.

The patient was off dopamine agonists therapy from 2000 to 2011, but due to a gradual increase in PRL level (Graph 1) associated with intense and frequent headaches, CAB was introduced in April 2011, normalizing her PRL level and leading to a reduction in headache. She experienced various physical side effects such as dizziness, vertigo, headache, nausea, vomiting, asthenia, constipation, alopecia, and edema, but no behavioral changes or mental symptoms. In 2019, a new MRI revealed an asymmetrical pituitary gland with diminished volume on the right side and encroachment of the suprasellar cistern, as well as a slight deviation of the infundibulum to the left. The optic chiasma was normal.

The initial dose of CAB was.5 mg per week, and the maintenance dose has been adjusted according to her test results over the years. Patient is currently taking two and a half pills or 1.25 mg weekly.

The patient has not reported behavioral changes or any mental complaint that impacted her daily routine so far. In 2020, she was invited for an interview with the clinical research project entitled “Physical and Behavioral Changes in Prolactinoma Patients Using Cabergoline” in partnership with the Clementino Fraga Filho University Hospital and the Institute of Psychiatry of the Federal University of Rio de Janeiro.

Patient reported no family history of substance abuse, psychiatric hospitalization, or suicide attempts. Her grandmother and son each had one previous episode of depression, but the patient had no such history herself. In her first psychiatric interview, performed for research purposes, she presented with normal physical appearance, cooperative, euthymic, with normal affect, slightly accelerated speech, regular thought process and content, normal cognition, and regular insight.

On the Mini International Neuropsychiatric Interview (MINI) (10) she was diagnosed with two previous mild depressive episodes and current binge eating disorder. She answered all the study’s standardized questionnaires: Young Mania Rating Scale (YMRS) (11), South Oaks Gambling Scale (SOGS) (12), Barratt Impulsiveness Scale (BIS11) (13), Compulsive Buying Scale (CBS) (14), Binge Eating Scale (BES) (15), Sexual Functional Questionnaire (SFQ) (16). She had significantly high scores on all three of the latter.

The patient scored 24 points on BES, considered moderate binge eating disorder. She described some unusual eating behaviors such as “eating without feeling hungry” and “eating just to chew food.” She even reported some event of vacuous chewing. These symptoms began in 2013 and remain at present. She gained approximately 18 kg over the years (weighting 78 kg in September 1998 and 96 kg at her last appointment in September 2020).

Patient scored 14 on CBS, considered impulsive buying behavior. She described three lifetime events: she bought 15 pairs of shoes in 2015, she began buying much more food to cook at home in 2016 and her relatives complained about it, and she reported buying jewelry on impulse (earrings, neckless, etc.), leading to financial problems.

Patient scored 94 points on BIS, considered high risk for impulsivity. On the other scales she did not score higher, but her sexual behavior stands out. Even though she did not score high in the SFQS, she described her sexuality as much more pronounced than before, with markedly increased libido and several daily episodes of masturbation.

**FIGURE 1:**
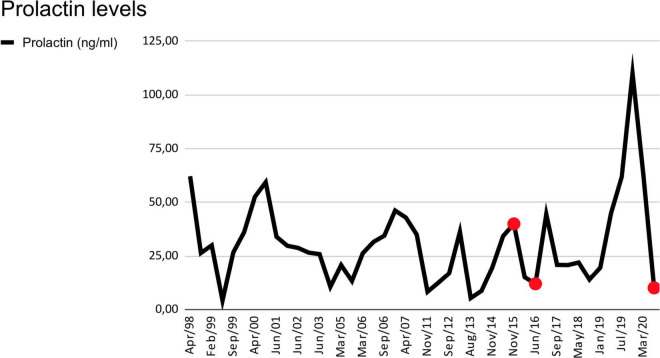
Prolactin levels are in ng/mL. Serum levels from April 1998, to March 2020. Normal levels are from 5 to 25 ng/mL. The red dots indicate moments of major impulsive behavior.

Prolactin variations in the period is shown in Figure 1.

## Diagnostic Assessment, Intervention, and Follow-Up

The patient was instructed to taper the dose of CAB, and despite her fear of worsening PRL control, she has been able to lower the dosage. She reports feeling well with this dose reduction, but sometimes the impulses to buy and eat reemerge. She has been accompanied by the Psychiatric Outpatient Clinic for psychological support and guidance. From the patient’s perspective, she presents personal, economic, and family problems. Her weight gain has generated conflict and irritability with her children. Currently (April–May 2021) she is experiencing binge eating of sweets, and her gastroesophageal reflux has worsened with her impulsive eating. She is in debt due to her impulsive shopping, which has caused various family conflicts.

## Discussion

Women with prolactinoma using dopamine agonists and with normal PRL levels present restored gonadal function in 80–90% of cases. These drugs recover their fertility and can conceive, regardless of tumor size (17). Pituitary tumors are classified as microadenomas (<1 cm) versus macroadenomas (⪖1 cm) (2).

The current patient with prolactinoma had no information on her initial tumor size and was already on dopamine agonists therapy (BRC was the available drug at the time). Her PRL level was 66.5 ng/ml (normal range 5–25 ng/dl). As reported, her treatment was irregular due to the clinical history of side effects from BRC, especially gastrointestinal effects. However, approximately one year after her diagnosis she became pregnant, leading to suspension of dopamine agonists as recommended by various authors (17–19).

The patient remained off medication for 11 years, which usually occurs in post-gestational periods in patients with prolactinoma (17, 18). However, she underwent annual monitoring, and her dopamine agonists therapy was resumed (this time with CAB) when her prolactin levels began to increase, and she presented altered pituitary imaging.

In the recent medical literature, the relationship between the use of dopamine agonists and impulse control disorders has been studied in patients with previous diagnosis of psychiatric diseases, as well as in healthy subjects (5–9, 20, 21). The initial focus was on patients with Parkinson’s disease, who usually receive high doses of dopamine agonists (21). However, ICD has been also identified in patients with prolactinoma, who often need lower doses of this class of medications. Such disorders can lead to personal, professional, and financial losses for these patients. They are frequently underdiagnosed by their endocrinologists or general practitioners.

Physiologically, the dopamine pathways consist of the nigrostriatal pathway, related to motor function, with D2 and D3 receptors; mesocortical and mesolimbic pathway, related to the reward system, with D3 receptors; and tuberoinfundibular pathway, which regulates PRL secretion, with D2 receptors. Dopamine agonists must thus act on the tuberoinfundibular pathway to promote its function in PRL control. However, dopamine agonists are not specific to one kind of receptor: they also bind to D1 and D3 receptors, which produces effects on the reward system. The hypothesis of ABCB1 gene polymorphisms would also explain the occurrence of ICD, influencing or altering the function of P-glycoprotein 1 (P-gp1), a transporter protein that takes substrates from the neuron and releases them into the bloodstream, potentially causing susceptibility to dopamine agonists side effects (22, 23).

The most widely studied and reported ICDs are hypersexuality, pathological gambling, compulsive shopping, and binge eating, and their prevalence in the general population is about 8% (24). Hypersexuality is more prevalent in men; the literature reports few cases in women: 3 with CAB or BRC and 1 with quinagolide, an ergot derivative also used to treat prolactinoma (25).

According to a review article published in 2019 on psychological effects in patients with prolactinoma or PRL-secreting adenoma, independently of previous report of psychiatric illness, patients on lower or higher doses of antidepressants presented ICDs and other psychoses, mania, and worsening of depression (5–9, 23, 26).

A case-control study selected 77 patients with prolactinoma and 70 patients with non-functioning adenoma from January 2001 to December 2011; 24.68% of prolactinoma patients had a positive ICD screening assessment for hypersexuality, punding, or pathological impulses to shop or gamble, with no statistical association between tumor size, type of dopamine agonist, or duration or dosage of the medication. ICD, more specifically hypersexuality, was significantly associated with male gender (7).

Several studies also reached similar conclusions, namely that ICD appears to be associated with male gender, but without evidence regarding age (5–9, 23, 25–27). Importantly, suspension of dopamine agonists therapy in most cases led to resolution or improvement of the psychiatric disorder. However, suspension of treatment may make PRL control more difficult, indicating the need for individual assessment of the feasibility of surgical treatment, maintenance of dopamine agonists therapy, and associated psychotherapy and/or antipsychotic medication such as aripiprazole.

Frequently, the patients are underdiagnosed by their endocrinologists or general practitioners because they do not ask about such effects either for the lack of knowledge of the ICD (and consequently they do not ask about it) or for the insecurity of reducing the medication.

## Patient’s Perspective

In our patient, the first symptoms of impulsivity appeared after three years of treatment with CAB, that is, less than the four-year criterion of time since onset of symptoms. The symptoms were completely new in her life, since she had no previous report of mania, hypomania, or any other impulsivity disorder. Unlike other patients treated with CAB, her side effects were nearly all physical. Her impulsivity impacted her daily life, with growing financial debt, weight gain, and decreased mood. The tools to identify these complex questions were thus impulsivity scales, which helped to describe all the impulsive symptoms.

The patient experienced a decrease in her impulsivity symptoms with the reduction in her CAB dosage, thereby corroborating the close relationship between dopamine agonists and the symptoms. After her psychiatric work-up she reported an improvement in her symptoms. Despite her initial resistance to dose reduction, the patient agreed to try tapering her medication, which we are doing progressively. The patient has reported improvement in her complaints, despite occasional urges to shop and overeat.

Prolactinoma patients on dopamine agonists do not usually voice behavior changes to their physicians, nor do they even realize what is happening. Therefore, it is extremely important to proactively investigate impulsivity symptoms, given the relevant evidence of the correlation between such symptoms and dopamine agonists use. Many patients may feel too shy to admit these symptoms. We also emphasize the importance of interdisciplinarity, given that in the current case the psychiatric work-up helped identify symptoms that had not been detect during the regular clinical examination.

Clinicians must therefore pay special attention and thoroughly observe patients in on-going CAB therapy, especially after four years or more on the medication. We recommend some possible interventions such as the use of impulsivity scales for measurement in asymptomatic patients, psychiatric evaluation in cases of evident impulsive behavior, and revision of the maintenance dose, if necessary, due to possible uncontrolled impulsiveness. It is also important to inform the patient about the potential side effects, including impulsivity.

## Data Availability Statement

The original contributions presented in the study are included in the article/supplementary material, further inquiries can be directed to the corresponding author.

## Ethics Statement

The studies involving human participants were reviewed and approved by Comitê de Ética do Hospital Universitário Clementino Fraga Filho - UFRJ. The patients/participants provided their written informed consent to participate in this study.

## Author Contributions

AC was the attending psychiatrist and reported on the patient’s psychiatric aspects. AA accompanied the patient under the supervision of AV. MB, JN, and RS collaborated in the literature review, writing, and publishing. MG contributed expertise to the project. AN supervised and reviewed all versions of the manuscript related to psychiatry. AV supervised AA, MB, JN, and RS in the patient’s clinical management and preparation of the article. All authors contributed to the article and approved the submitted version.

## Conflict of Interest

The authors declare that the research was conducted in the absence of any commercial or financial relationships that could be construed as a potential conflict of interest.

## Publisher’s Note

All claims expressed in this article are solely those of the authors and do not necessarily represent those of their affiliated organizations, or those of the publisher, the editors and the reviewers. Any product that may be evaluated in this article, or claim that may be made by its manufacturer, is not guaranteed or endorsed by the publisher.
